# Linezolid Is Associated with Serotonin Syndrome in a Patient Receiving Amitriptyline, and Fentanyl: A Case Report and Review of the Literature

**DOI:** 10.1155/2013/617251

**Published:** 2013-03-04

**Authors:** Lampros Samartzis, Paraskevi Savvari, Sofoklis Kontogiannis, Stavros Dimopoulos

**Affiliations:** ^1^Department of Psychiatry, Mental Health Services, Athalassa Psychiatric Hospital, 1452 Nicosia, Cyprus; ^2^Department of Clinical Therapeutics, National and Kapodistrian University of Athens, Alexandra Hospital, 80 Vas. Sofias Avenue, 11528 Athens, Greece

## Abstract

We report a unique case of an adverse interaction between the oxazolidinone antibiotic linezolid, the tricyclic antidepressant amitriptyline and the opioid analgesic fentanyl in a 68-year-old woman with advanced ischemic peripheral arterial disease and sepsis, under empirical antibiotic treatment. We also summarize the current relevant literature as identified via PubMed, EMBASE, and PsycINFO as well as reference sections of selected articles.

## 1. Case

Ms. B, a 68-year-old woman, presented at our outpatient clinic with intense lower foot pain and fever since a week. Clinical examination revealed the 3 first phalanges of the left foot painful, cyanotic, and swollen in the absence of palpable pulsus at the ventral and dorsal tibial arteries with concomitant fever (38.5°C) and tachycardia (110 bpm). A complete blood count showed elevated total number of white blood cells (21 × 10^9^/L), consisted of 93% from neutrophil granulocytes. She was admitted to our hospital due to sepsis and possible diagnosis of infection of the ischemic left foot. Anamnestic history included advanced peripheral ischemic disease, diabetes mellitus type II, arterial hypertension, and major depression. The patient was receiving treatment with fentanyl transdermal patch 25 *μ*g/h every 72 h since 10 days and amitriptyline 25 mg BD for depression. The low dose of amitriptyline 25 mg BD was maintained due to its antidepressant [1, 2] as well as analgesic effects on chronic pain [3–5] and especially painful diabetic limb [6–12]. During her stay in the medical ward, she was treated with empirical antibiotics including cloxacillin, 3rd generation beta-lactams, and aminoglycosides with an initial general improvement. However, at the 10th day patient had a new onset of high fever (38.7°C) and linezolid 600 mg every 12 hours was added to the treatment regimen and cloxacillin was stopped. Within the first 24 hours of antibiotic change treatment, the patient had a rapid clinical deterioration manifesting symptomatology of restlessness, diaphoresis, tremor, shivering, myoclonus, and high fever (40°C), as well as gradual mental status disorders with disorientation, confusion, and coma. The patient was intubated due to severe respiratory difficulties according to the criteria of our clinic, and transferred to the intensive care unit. Brain computerized tomography and lumbar puncture (LP) for the exclusion of central neural system (CNS) infection were unremarkable. The constellation of the above neurological and mental state features in the presence of serotonergic medication [13–15] and the abstinence of other CNS pathology leads to the diagnosis of serotonin syndrome according to the diagnostic criteria of Hunter [16, 17] and Sternbach [14]. The first signs of improvement appeared a few hours after the interruption of linezolid and amitriptyline. Withdrawal of sedation and ventilator weaning took place 48 hours later. The patient gradually regained her consciousness and orientation to person, location, and time, as expected in the opposite order in which she lost orientation in the beginning of the confusion state [18]. 

Amitriptyline is a tricyclic antidepressant (TCA), a drug category that is believed to act through boosting of serotonin and norepinephrine neurotransmission via blockade of serotonin and norepinephrine reuptake pumps [19], as well as via desensitizing both 5-HT_1A_ and beta-adrenergic receptors. Tertiary amineTCAs, such asamitriptyline, imipramine, and clomipramine, are more potent inhibitors of serotonin reuptake than secondary amine TCAs, such as nortriptyline or desipramine, therefore theoretically more prone to be involved in the development of serotonin syndrome.

Fentanyl is an synthetic opioid analgesic, which is characterised by high lipid solubility and therefore it easily penetrates the central nervous system (CNS), where it acts through binding to *μ*-opioid receptors (mu receptors) resulting in inhibition of pain neurotransmission [20–22]. Fentanyl belongs to the phenylpiperidine subcategory of opioid substances, as do methadone, pethidine (meperidine), tramadol, propoxyphene, and dextromethorphan. Phenylpiperidine opioids are considered to have mild serotonin-reuptake inhibition (SRI) properties and therefore a higher possibility, for involvement in serotonin syndrome development [23]. The nonphenylpiperidine opioids, such as buprenorphine, codeine, morphine, and oxycodone, were not reported to show SRI properties [24]. Interestingly, there is a report of paradoxical reaction regarding fentanyl use in the treatment of serotonin syndrome [25], which had been induced by coadministration of the selective serotonin reuptake inhibitor (SSRI) fluoxetine and the reversible inhibitor of monoamine oxidase A (MAO-A) moclobemide. 

Linezolid is an oxazolidinone category antibiotic that is believed to act through early inhibition of protein synthesis via binding to the 23S portion of the 50S ribosomal bacterial rRNA subunit [26, 27] inducing conformational structural changes and preventing tRNA to enter and functionally bind to the ribosome [28] therefore inhibiting mRNA translation. Linezolid is a totally synthetic compound that was initially synthesized as a reversible MAO inhibitor class antidepressant [29].

Serotonin syndrome usually consisted of a constellation of neurological and mental state symptoms and commonly diagnosed according to the widely accepted criteria of Sternbach and/or Hunter [14, 16, 17, 30], as summarised in Table 1. Symptoms usually improve with the withdrawal of the predisposing drug agents plus supportive care, as there is no specific evidence-based treatment of the syndrome [31]. Cyproheptadine is a H_1_ histamine receptor antagonist as well as a nonspecific serotonin receptor antagonist [32] which may have a role in serotonin syndrome treatment in a usual dose of 8 mg via the nasogastric tube.

The development of the serotonin syndrome has been reported as an interaction of linezolid plus almost every category of antidepressant medication. Interactions have been found with the selective serotonin reuptake inhibitors (SSRIs) as citalopram [29, 33–39], escitalopram [34], paroxetine [40], fluoxetine [41, 42], sertraline [43–45], with tryptophan, a precursor of serotonin [46], with noradrenaline and specific serotonergic agents (NaSSA) as mirtazapine [29], with serotonin 2 antagonist/reuptake inhibitor (SARI) as trazodone [36], the azapirone category anxiolytic buspirone that is a serotonin 1A partial agonist and serotonin stabilizer [47], the dual serotonin and noradrenaline reuptake inhibitors (SNRIs) as the antidepressants venlafaxine [36, 48–51] and duloxetine [52], and the tricyclic antidepressant (TCA) imipramine [49]. Although amitriptyline [14, 53–56] and fentanyl [57–64] have been both involved in serotonin syndrome, no case has been reported involving their combination. 

To the knowledge of the authors, this is the first case of serotonin syndrome reported in a patient receiving this common three-drug combination.

Fentanyl is primarily metabolized via CYP-P450-3A4 isoenzyme system by oxidative dealkylation to the main metabolite norfentanyl which is inactive [65].

Amitriptyline is metabolised via CYP-P450-1A2 isoenzyme by demethylation to form the active metabolite nortriptyline [66] and via CYP-P450-2D6 by hydroxylation to form inactive metabolites [19], but there is also data showing that demethylation via CYP-P450-3A4 plays an important role in its metabolism [67–69]. Therefore a pharmacokinetic interaction between fentanyl and amitriptyline could not be excluded. Nevertheless, in our case, coadministration of amitriptyline and fentanyl did not reveal any symptomatic interaction, as the serotonin syndrome was induced only after the addition of linezolid to the treatment regimen. 

Linezolide is metabolised via oxidation procedure in a way independent of cytochrome P450 (CYP-450); consequently there is no possible pharmacokinetic mechanism of interaction between linezolide and other medication metabolized through CYP450 pathways [27].

Also, the serotonin syndrome pathophysiological mechanism does not include idiosyncratic, neither idiopathic nor pharmacokinetic drug reactions, but it is considered to be a predictable and preventable pharmacodynamic consequence of the excess of serotonergic agonism in CNS and peripheral serotonergic receptors [17].

We suggest that physicians avoid use of linezolid in patients receiving combination of amitriptyline and fentanyl due to possible serotonin syndrome induction.

## Figures and Tables

**Table 1:** Widely accepted diagnostic criteria for serotonin syndrome.

Criteria of Hunter, 2003 [16, 17].	
Presence of a serotonergic agent and 1 of 5	
(1) Spontaneous clonus	
(2) Inducible clonus and agitation or diaphoresis	
(3) Ocular clonus and agitation or diaphoresis	
(4) Tremor and hyperreflexia, or	
(5) Hypertonia and temperature >38°C and ocular clonus or inducible clonus	
Criteria of Sternbach, 1991 [14, 30].	
Presence of all of the following	
(a) Recent addition or increase in a known serotonergic agent	
(b) Absence of other possible aetiologies (infection, substance abuse, substance withdrawal, etc.)	
(c) No recent addition or increase of a neuroleptic agent	
(d) At least 3 of the following 10	
(1) Mental status changes (confusion, hypomania)	
(2) Agitation	
(3) Myoclonus	
(4) Hyperreflexia	
(5) Diaphoresis	
(6) Shivering	
(7) Tremor	
(8) Diarrhoea	
(9) Incoordination	
(10) Fever	
